# Full-length transcriptome of *Misgurnus anguillicaudatus* provides insights into evolution of genus *Misgurnus*

**DOI:** 10.1038/s41598-018-29991-6

**Published:** 2018-08-03

**Authors:** Shaokui Yi, Xiaoyun Zhou, Jie Li, Manman Zhang, Shuangshuang Luo

**Affiliations:** 1 College of Fisheries, Key Lab of Freshwater Animal Breeding, Ministry of Agriculture, Huazhong Agricultural University, Wuhan, 430070 P.R. China; 2 Fish Genetics and Breeding Laboratory, the Ohio State University South Centers, Piketon, 45661 USA

## Abstract

Reconstruction and annotation of transcripts, particularly for a species without reference genome, plays a critical role in gene discovery, investigation of genomic signatures, and genome annotation in the pre-genomic era. This study generated 33,330 full-length transcripts of diploid *M*. *anguillicaudatus* using PacBio SMRT Sequencing. A total of 6,918 gene families were identified with two or more isoforms, and 26,683 complete ORFs with an average length of 1,497 bp were detected. Totally, 1,208 high-confidence lncRNAs were identified, and most of these appeared to be precursor transcripts of miRNAs or snoRNAs. Phylogenetic tree of the *Misgurnus* species was inferred based on the 1,905 single copy orthologous genes. The tetraploid and diploid *M*. *anguillicaudatus* grouped into a clade, and *M*. *bipartitus* showed a closer relationship with the *M*. *anguillicaudatus*. The overall evolutionary rates of tetraploid *M*. *anguillicaudatus* were significantly higher than those of other *Misgurnus* species. Meanwhile, 28 positively selected genes were identified in *M*. *anguillicaudatus* clade. These positively selected genes may play critical roles in the adaptation to various habitat environments for *M*. *anguillicaudatus*. This study could facilitate further exploration of the genomic signatures of *M*. *anguillicaudatus* and provide potential insights into unveiling the evolutionary history of tetraploid loach.

## Introduction

Transcriptome analysis is a powerful tool for uncovering the relationships between genotype and phenotype, leading to a better understanding of the underlying pathways and genetic mechanisms controlling cell growth, development, immune regulation, etc^1^. The complexity of transcriptome determines the reliance on high-throughput tools for transcriptome analysis. Thus RNA sequencing (RNA-seq) technique has rapidly become an important tool in biological studies. As a high-throughput method to investigate differential expression of genes, RNA-seq also promised an unprecedented sensitivity for detecting the rare transcripts, splice variants and small RNAs.

Recently, the development of RNA-seq technology has strongly facilitated the application to transcriptomic studies of fish species^2–4^. Searching the Web of Science (Thomson Reuters) database for the terms ‘RNA-seq AND fish’ returns 13,700 articles published in the period of 2008–2017, with 3,480 articles published in 2017. Although RNA-seq has been applied to large numbers of fish genetic studies involved with various fish species, the differences in transcript abundance and the presence of different isoforms greatly challenge the assembly of a transcriptome from short reads, such as those yielded from Illumina or Ion Torrent sequencing platforms. To date, most second-generation high-throughput sequencing platforms offer a read-length which is shorter than the typical length of a eukaryotic mRNA, including a methylated cap at the 5′ end and poly-A at the 3′ end. With the advance of third-generation sequencing technology, the length of sequencing reads has been dramatically increased^5^. For instance, the average read length of PacBio (Pacific Bioscience) single molecule real-time (SMRT) sequencing is around 10 kb and subreads can reach lengths of up to 35 kb with the PacBio Sequel™ system (PacBio, Menlo Park, CA, USA)^6,7^. Due to the much longer read lengths of the third-generation sequencing, the precise location and sequence of repetitive regions and isoforms can often be resolved within a single read.

*Misgurnus anguillicaudatus*, also known as oriental weatherloach, is distributed throughout China and mainly occurs in the Yangtze River basin^8,9^. Due to its superior nutritional values and excellent flavor, this famous loach is widely consumed and favored in East Asia region. Therefore, *M*. *anguillicaudatus* has become an important freshwater aquaculture species for several decades in China^10^. However, the genome and gene set of this species is still not available due to the high heterozygosity levels and complex polyploidy. Thus a full-length transcriptome is quite essential for documenting the genomic signatures of *M*. *anguillicaudatus*.

This study firstly proposed the full-length transcriptome of *M*. *anguillicaudatus* derived from 12 different tissues using the PacBio SMRT sequencing that could be used as a reference in some cases. Moreover, the annotation of *M*. *anguillicaudatus* gene set could improve the whole genome annotation, contribute towards better understanding of the complexity of the genome and serve as reference sequences for gene functional studies. Although previous studies^10–13^ have provided important insights into the evolutionary status and adaptation of *M*. *anguillicaudatus*, little information is available on the difference of evolutionary adaptations of regular diploid and tetraploid *M*. *anguillicaudatus*^10^. Therefore, a number of full-length transcripts of *M*. *anguillicaudatus* obtained in this study were also applied to evaluate the evolutionary status and adaptive evolution of diploid and tetraploid *M*. *anguillicaudatus* which would provide novel insights into the genetic characteristics of natural polyploidy.

## Results

### SMRT sequencing, completeness and Functional annotation

**Figure 1:**
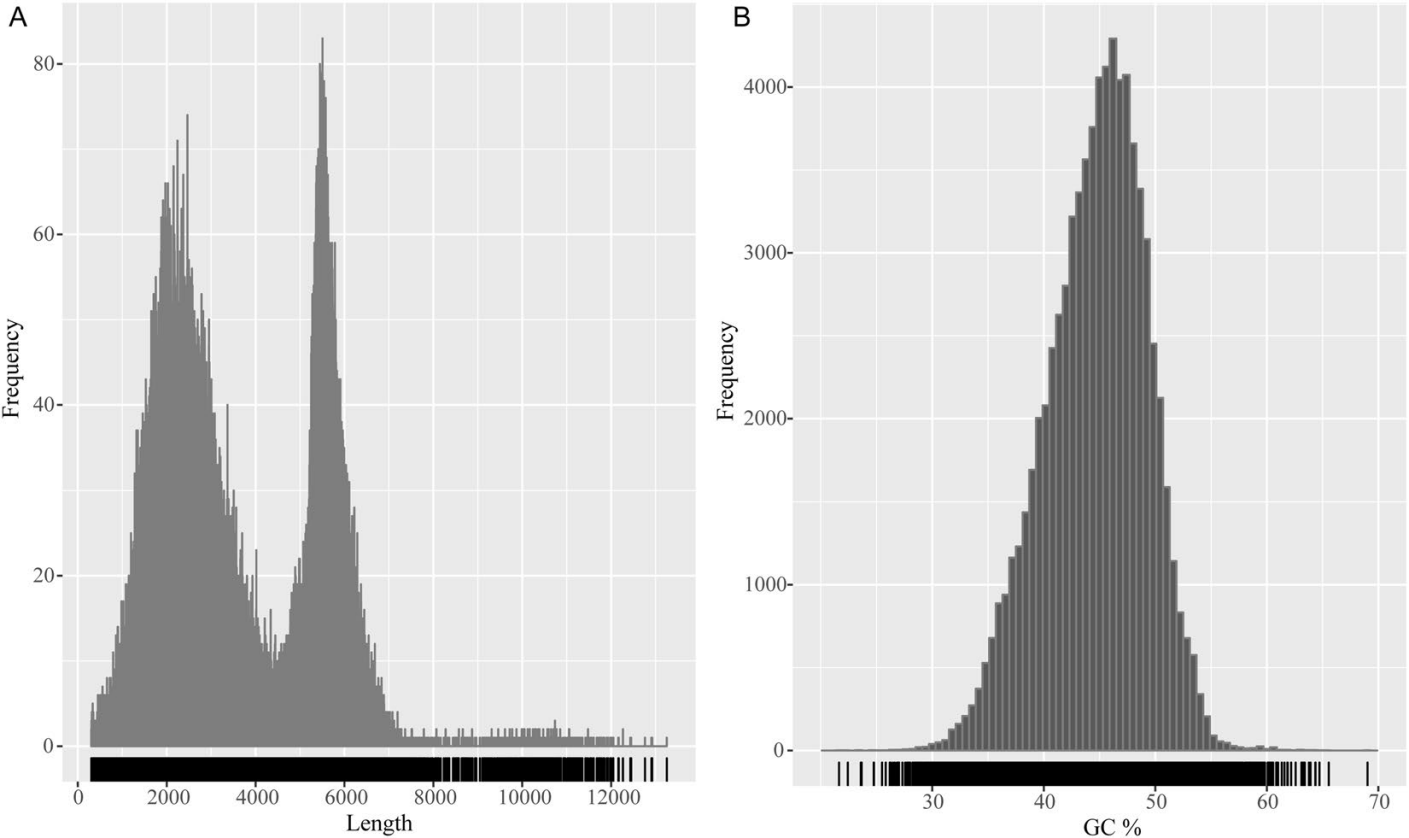
The frequency distribution of the length (**A**) and GC contents (**B**) of the 76,787 consensus sequences.

To obtain a representative full-length transcriptome for diploid *M*. *anguillicaudatus*, two size fractions (1–5 kb and 4.5–10 kb) of sample were sequenced on two SMRT cells using PacBio Sequel system (Table 1). Sequence data were processed through the Pacbio Iso-Seq pipeline. A total of 1,149,167 reads of insert (ROIs), including 627,338 ROIs from a SMRT cell for 1–5 kb fractions and 521,829 ROIs from another SMRT cell for 4.5–10 kb fractions, were generated with an average of 3,468 bp in length. Non-Chimeric ROIs sequences were filtered into two groups of sequences comprised of full-length ROIs sequences and non-full-length ROIs sequences. Only the reads with two primers and poly-A tail were classified to full-length non-chmeric reads, and 422,274 full-length non-chmeric reads were remained after circular consensus sequence (CCS) generation and filtering for full-length read classification. These FL reads were clustered into consensus clusters based on Quiver principle, and then 135,316 high-quality (HQ) and consensus isoforms were merged into 76,787 final consensus sequences in total. The length of these consensus sequences ranged from 302 bp to 13,253 bp with an average length of 3,614 bp, and the length exhibited a bimodal distribution as expected (Fig. 1A). The transcript N50 was 5,234 bp, and the average GC content was 41.4% (Fig. 1B).

**Table 1:** The PacBio SMRT sequencing information of *M*. *anguillicaudatus.*

Sample name	cDNA size (kb)	Reads of Insert	Read bases of insert (bp)	Mean read length of insert (bp)	Mean read quality of insert	Mean number of passes	full-length non-chimeric reads
2n_MA	1–5	627,338	2,192,131,499	3494	0.88	3	220,682
2n_MA	4.5–10	521,829	1,795,489,735	3441	0.89	3	201,598

Subsequently, we utilized CD-HIT-EST for further clustering to obtain the non-redundant transcripts and obtained a total of 33,330 full-length transcripts of *M*. *anguillicaudatus*. BUSCO (Benchmarking Universal Single Copy Orthologs) was utilized to determine completeness of our transcript dataset with the Actinopterygii dataset (http://busco.ezlab.org/v2/datasets/). The results showed that this dataset contains 65.6% complete and 10.5% partial BUSCO orthologues. Meanwhile, we also used an alternative method, CEGMA (Core Eukaryotic Genes Mapping Approach) to evaluate the transcripts completeness and found that 83.9% (208 genes) of the 248 core CEGMA genes are complete in this dataset.

**Figure 2:**
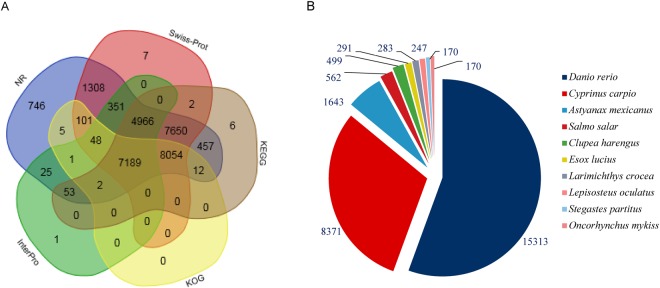
Functional annotations of the non-redundant transcripts with the public databases. (**A**) Venn diagram of the annotation between NR, COG, KEGG, Swiss-Prot and InterPro databases. (**B**) The distribution of homologous species annotated in the NR database.

Functional annotation of the non-redundant transcripts was investigated using 6 different protein public databases (Fig. 2A). Using BLASTX, 30,968 sequences (92.9%) were successfully annotated based on the NR (NCBI non-redundant proteins) database. The transcripts were then classified using the Swiss-prot (a manually annotated, non-redundant protein database), InterPro (https://www.ebi.ac.uk/interpro/), COG (Clusters of Orthologous Groups) and KEGG (Kyoto Encyclopedia of Genes and Genomes) databases (Supplementary Table S1). Of all the hits to the NR proteins from BLASTX, the annotation results showed that the majority of the transcripts closely matched the genes from other fish species. The transcripts had the highest number of hits to the *Danio rerio* (15,313 hits, 45.9%), as expected, followed by *Cyprinus carpio* (8,731 hits, 26.2%) and *Astyanax mexicanus* (1,643 hits, 4.9%) proteins (Fig. 2B). Notably, 4.5% of transcripts showed no homologous sequences in the public databases.

### Prediction of gene families and coding sequences

**Figure 3:**
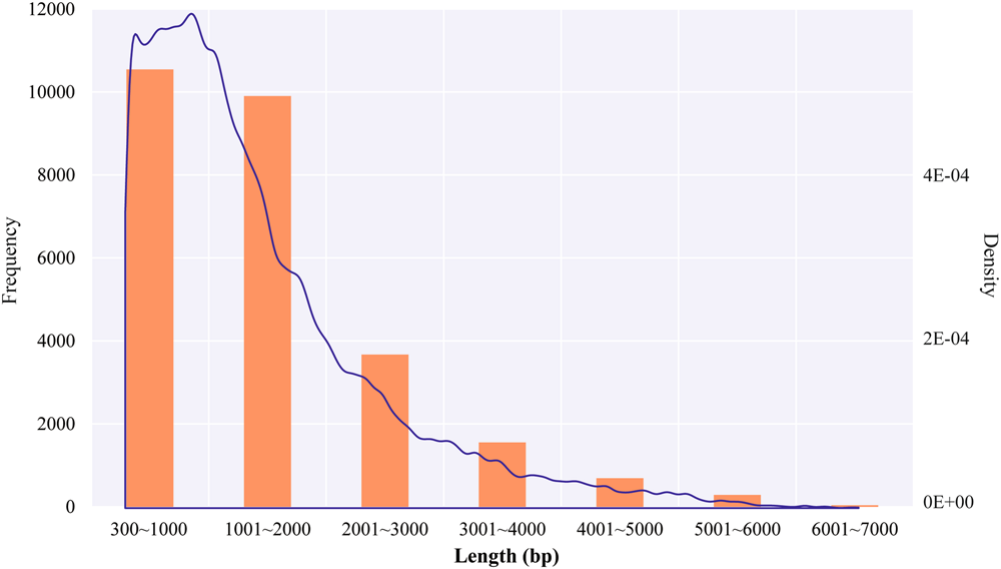
Length distribution of the complete ORFs predicted with TransDecoder.

We utilized the COGENT to further partition these non-redundant transcripts into putative gene families and reconstruct each family into one or several full-length unique transcript models. Of the 33,330 transcripts, COGENT constructed 6,918 gene families with two or more isoforms (23,894 transcripts in total). A total of 26,683 complete ORFs (Open Reading Frames) were identified with TransDecoder (Supplementary Table S2). The lengths of the complete coding sequences ranged from 300 to 6,867 bp with an average length of 1497 bp (Fig. 3). Among the 26,683 complete ORFs, a minority of ORFs (1,006, 3.8%) showed no homologous entry in Pfam database.

### Analysis of candidate long non-coding RNAs

**Figure 4:**
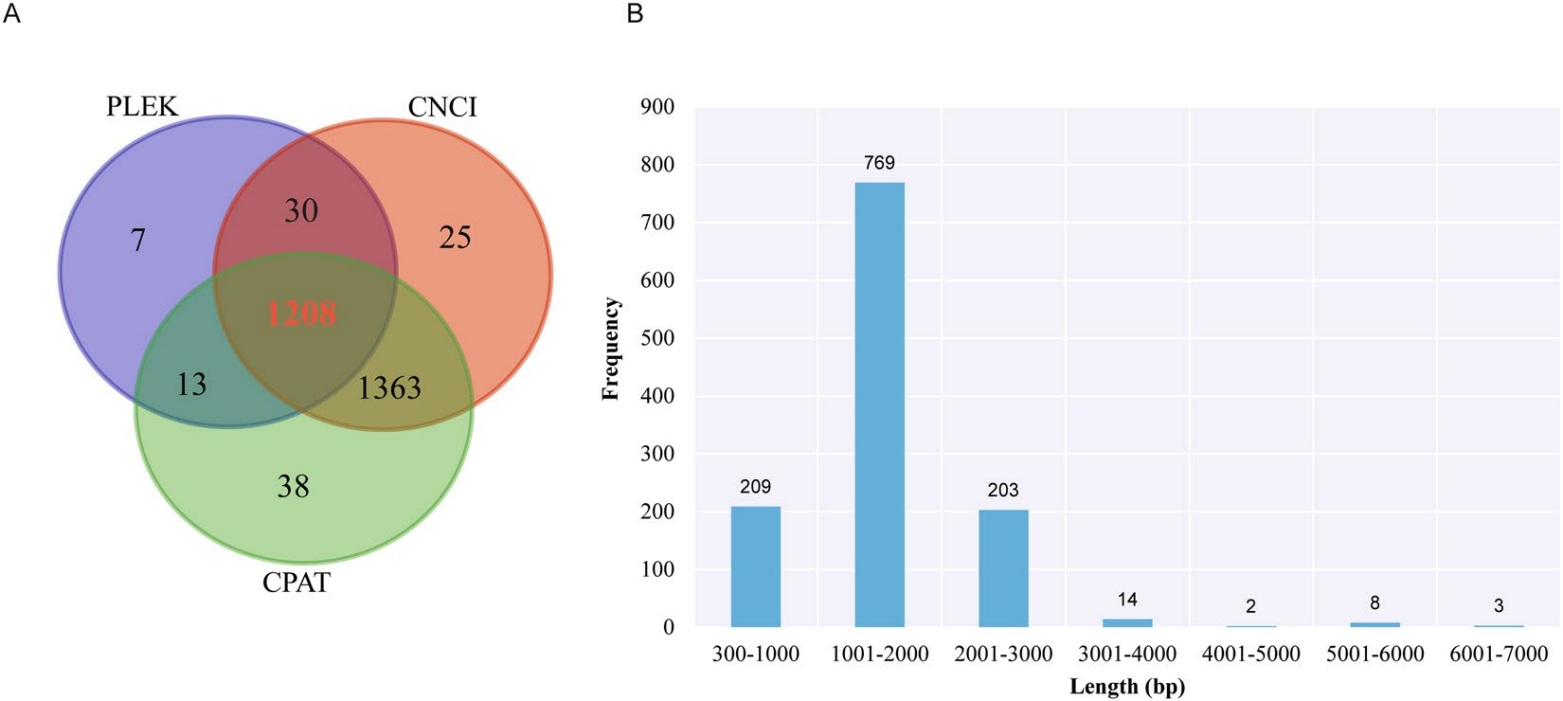
Prediction of lncRNAs in *M*. *anguillicaudatus*. (**A**) Venn diagram of the prediction with three methods. (**B**) Length distribution of the 1,208 predicted lncRNAs.

In addition to protein-coding RNAs, non-coding RNAs, which are attributed to poly-A non-coding RNAs, constitute a major component of the transcriptome. After annotation with the public databases, 2,776 non-annotated transcripts were remained. The number of lncRNAs predicted by each prediction method was shown in Fig. 4A. In total, 1,208 high-confidence lncRNAs were identified with an average length of 1,593 bp (Fig. 4B). Of these lncRNAs, only a minority (105 lncRNAs) was annotated with known RNA motifs by searching against Rfam ncRNA families^14^, and most of these appeared to be precursor transcripts of miRNAs or snoRNAs (Supplementary Table S3). The functions of the lncRNAs, especially the unannotated ones, need to be further verified.

### Identification of orthologous genes among *Misgurnus* species and phylogenetic tree

**Figure 5:**
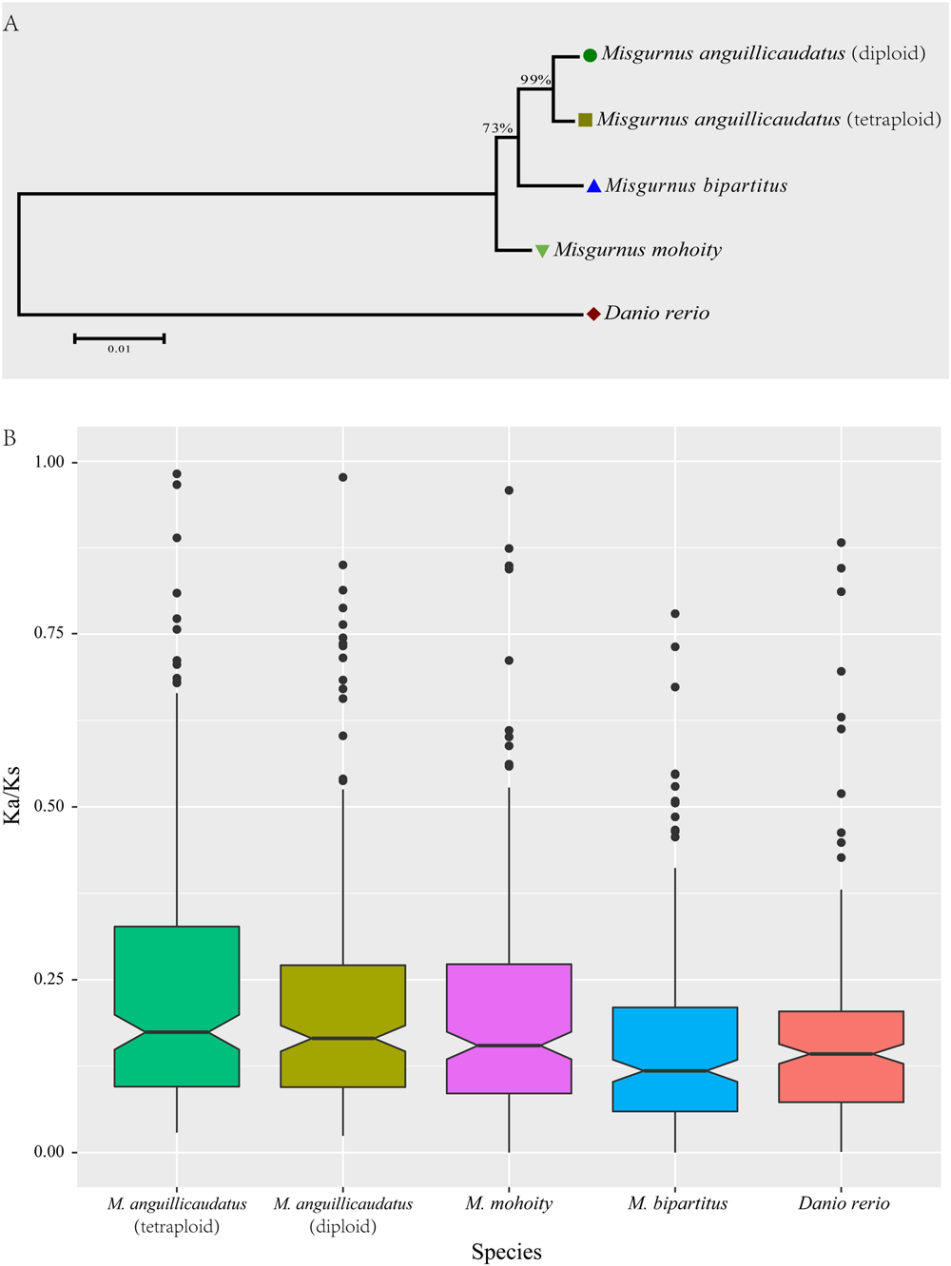
Phylogenetic tree of *Misgurnus* species inferred with RAxML (**A**) the substitution rates of *Misgurnus* species calculated with free-ratio model in codeml program (**B**).

To reveal the adaptive evolution of *Misgurnus* species respond to the specific environments they inhabit, a total of 1,918 one-to-one single-copy orthologous genes, ranging from 150 to 5796 bp in length, were identified among four *Misgurnus* species together with a outgroup *Danio rerio*. Subsequently, the alignments were trimmed with Gblocks. Totally, 1,905 single copy orthologous gene with length >150 bp were remained for the further analysis (Supplementary Table S4). Phylogenetic tree was inferred from the concatenated sequences of 1,905 orthologues using ML (Maximum Likelihood) method. The phylogenetic tree showed that diploid and tetraploid *M*. *anguillicaudatus* formed a clade with a bootstrap value of 99%, indicating a close evolutionary relationship (Fig. 5A).

### Accelerated Evolution and positively selective genes in *M*. *anguillicaudatus* clade

To assess the overall evolutionary rates within four *Misgurnus* lineages, the calculation of substitution rates (Ka and Ks) for each orthologues was performed with codeml using the free ratio model. The levels of the Ka/Ks ratios revealed a declining trend in the four *Misgurnus* lineages, with the tetraploid *M*. *anguillicaudatus* lineage revealing highest Ka/Ks ratios than the other three lineages (Fig. 5B), which indicates accelerated evolution occurred in tetraploid *M*. *anguillicaudatus* compared with other *Misgurnus* species. The positively selective genes (PSGs) were identified with branch site model using the diploid and tetraploid *M*. *anguillicaudatus* clade as foreground branch.

**Figure 6:**
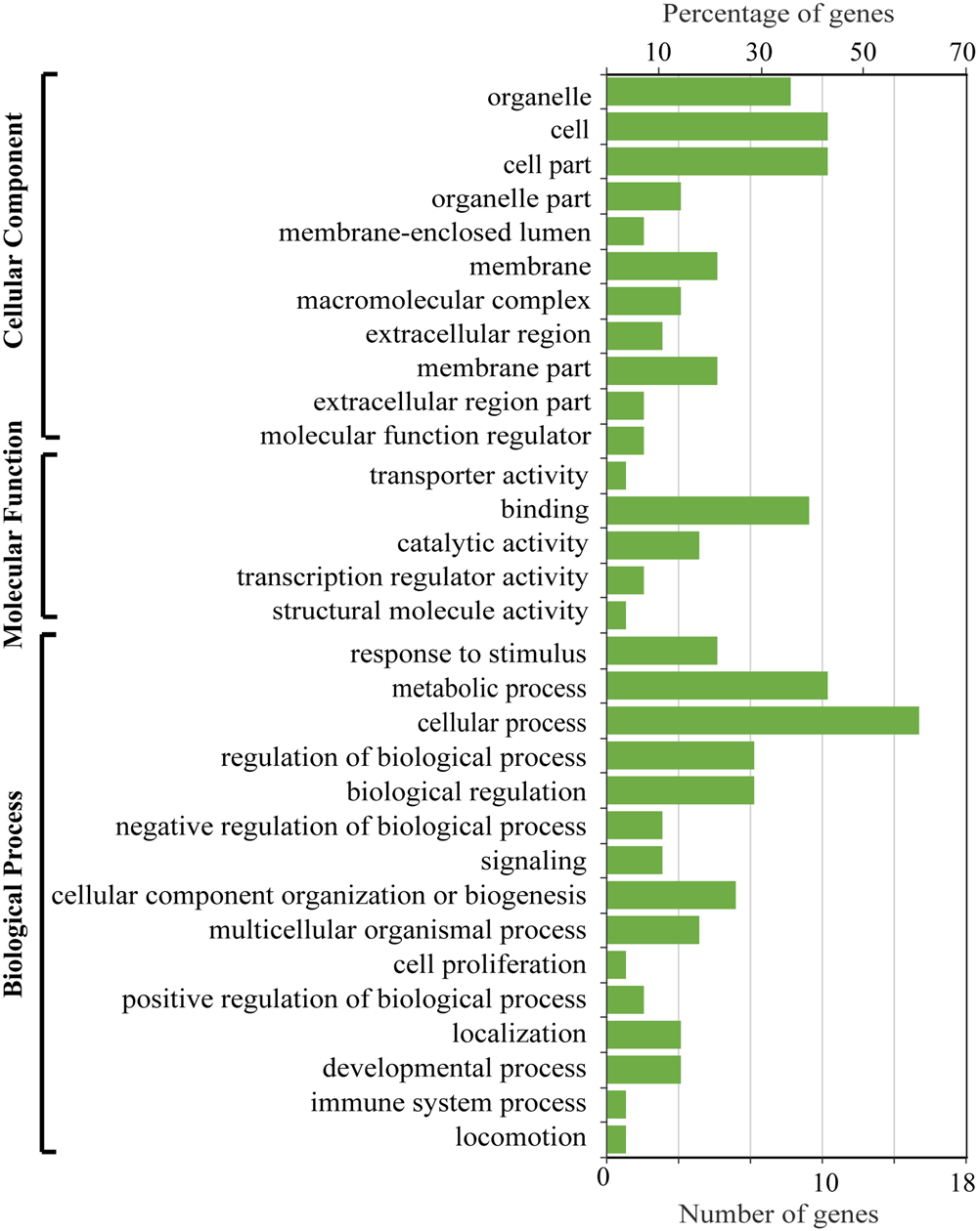
Distribution of GO classifications of positively selected genes in the diploid and tetraploid *M*. *anguillicaudatus* clade.

We used the branch-site model implemented in codeml to identify the positively selected genes (PSGs) and the positive selected sites (PSSs) in codons along the *M*. *anguillicaudatus* clade. We identified 28 PSGs in the diploid and tetraploid *M*. *anguillicaudatus* clade (Supplementary Table S5). We classified the PSGs by GO categories and plotted the distribution of GO classifications of PSGs among the GO categories (Fig. 6). GO categories “cellular processes”, “metabolic process”, “cell”, “cell part”, “binding” contained the largest percentage of genes. The GO enrichment results exhibited that these PSGs were significantly enriched in “regulation of body fluid levels” (*P* = 0.012), “polysaccharide binding” (*P* = 0.012) and “pattern binding” (*P* = 0.012), indicating that the PSGs play critical roles in these biological functions. Meanwhile, the PSSs were calculated using likelihood ratio tests (LRTs) with false discovery rate (FDR) correction (*P* < 0.05) and Bayes Empirical Bayes (BEB) estimates (BEB > 0.95). Nine PSGs with 10 positively selective sites were detected in the *M*. *anguillicaudatus* clade (Table 2). Among these PSSs, two sites in Ramp2, which is involved in protein transporter activity, were identified in the diploid and tetraploid *M*. *anguillicaudatus* clade, indicating that it plays important roles in environmental adaptations of *M*. *anguillicaudatus*.

**Table 2:** The positively selected sites identified in *M*. *anguillicaudatus* clade.

Orthologous ID	Site position	BEB probability	Gene name	Gene description
OG0102	362 R	0.991**	*WASHC4*	WASH complex subunit 4
OG0501	290 N	0.984*	*ncl*	nucleolin
OG0759	312 R	0.985*	*rpa1*	replication protein A1
OG0884	47 D	0.962*	*lipg*	lipase, endothelial
OG1052	72 V 94 Y	0.958* 0.982*	*ramp2*	receptor (G protein-coupled) activity modifying protein 2
OG1178	240 T	0.971*	*zgpat*	zinc finger, CCCH-type with G patch domain
OG1413	7 T	0.950*	*naf1*	nuclear assembly factor 1 homolog (S. cerevisiae)
OG1652	113 V	0.979*	*sf3b4*	splicing factor 3b, subunit 4
OG1728	87 S	0.982*	*ier2a*	immediate early response 2a

## Discussion

### Long read reference reconstruction of the full-length transcripts

Transcriptome reconstruction and annotation, particularly for species without a reference genome, has improved significantly with the development of sequencing techniques, and plays a critical role in gene discovery, deep exploration of genomic signatures, and genome annotation in the pre-genomic era^15^. In recent years, traditional short-read RNA-seq has been widely applied in the studies of investigating the RNA expression patterns although most of the transcripts assembled with the assemblers using short reads, such as Trinity^5^, SOAPdenovo-Trans^16^ or Velvet^17^, were not full-length (<1000 bp). However, short-read RNA-seq techniques still have some limits in precise reconstruction and reliable expression investigation of transcriptomic isoforms due to the complexity of the alternative splicing mechanism of eukaryotic cells, particularly in species without a reference genome sequence. In contrast, for a genome-free species, PacBio Iso-Seq is a superior strategy for the direct generation of a comprehensive transcriptome with accurate alternative splicing isoforms and novel genes. The advantages of SMRT sequencing have been extensively documented in previous studies. In this study, a full-length transcriptome of *M*. *anguillicaudatus* was sequenced with Iso-Seq technique and comprehensively analyzed. Our results showed that the non-assembled transcripts from Iso-Seq (Transcript N50 of 5,234) were much longer than the assembled transcripts from Illumina sequencing platforms (Unigene N50 of 1,662 bp)^12^, and many Iso-Seq sequencing subreads contained full-length ORFs.

In this study, we generated 33,300 transcripts using the PacBio Iso-Seq, which was much smaller than 81,300 transcripts *de novo* assembled in Luo *et al*.^18^ and showed high-efficiency in recovering full-length transcript. Even though the number of predicted ORFs in the PacBio dataset was less than that in the *de novo* dataset, the full-length transcripts included longer coding transcripts (26,683 ORFs with average length of 1457 bp) while the average length of ORFs in assembled transcripts was only 661 bp in the previous RNA-seq study^12^ of *M*. *anguillicaudatus*. The CEGMA/BUSCO completeness alignment of full-length transcripts indicated that our dataset contained expected core conserved genes, and indirectly revealing that only a minority of the genes was missing in the full-length transcripts.

Many transcriptome studies of *M*. *anguillicaudatus* based on Illumina platform have been reported^12,18–20^. Compared with those studies, our study produced a more comprehensive transcriptome data set with several features. First, more accurate full-length (with 22,683 completed ORFs) transcripts were generated, providing valuable information for detection of gene structure and variations and abundant sequences that can be directly used in the genetic functional studies without additional PCR cloning. Second, a total of 1,208 lncRNAs of *M*. *anguillicaudatus* were identified, and these lncRNAs could provide a useful resource for investigating the potential functions of lncRNAs in *M*. *anguillicaudatus* although only a minority of lncRNAs was annotated with known RNA motifs in Rfam database. Third, the full-length transcripts produced in this study would be used as reference to improve genome assembly and gene annotation of *M*. *anguillicaudatus*. Finally, the reliable ORFs and proteome of *M*. *anguillicaudatus* were essential to the identification of orthologous genes, which contribute to better understanding of the evolutionary status and adaptive evolution of diploid and tetraploid *M*. *anguillicaudatus*.

### Evolutionary status and positive selection in *Misgurnus* linages

In recent years, the extensive ploidy variability of *M*. *anguillicaudatus* is a hot research topic because it has several natural ploidy types in nature, including diploid^10^ (2n = 50), triploid (3n = 75), tetraploid^21^ (4n = 100), pentaploid^22^ (5n = 125) and hexaploid^23^ (6n = 150). Notably, most of natural *M*. *anguillicaudatus* individuals are diploid and tetraploid in China, and triploid, pentaploid and hexaploid are rare in nature. The complexity of ploidy brought many challenges in genetic or genomic studies of *M*. *anguillicaudatus*, and the polyploid loaches were often absent in the previous genetic studies of *M*. *anguillicaudatus*^18–20^. It’s quite urgent to unveil the evolutionary history and genomic signatures of polyploid loaches so that we could proceed into post-genome era of *M*. *anguillicaudatus*. In this study, we firstly investigated the evolutionary status of tetraploid *M*. *anguillicaudatus* in genus *Misgurnus*. The results exhibited that the tetraploid and diploid *M*. *anguillicaudatus* grouped into one clade, which coincides with the results inferred with mitochondrial genome^10^. *M*. *bipartitus* showed closer relationship with *M*. *anguillicaudatus* clade rather than *M*. *mohoity*, and the same topological relationships of three *Misgurnus* species were observed in the previous study^12^.

In genus *Misgurnus*, only *M*. *anguillicaudatus* is widely distributed in both north and south China, indicating that *M*. *anguillicaudatus* has strong adaptation to the various environments. The overall evolutionary rates of the *Misgurnus* lineages showed that tetraploid *M*. *anguillicaudatus* exhibited accelerated evolution compared with those of diploid *M*. *anguillicaudatus* and *M*. *mohoity*, indicating a trend of increase in the evolutionary pressure in the tetraploid *M*. *anguillicaudatus*. The function classifications of PSGs identified from the *M*. *anguillicaudatus* clade revealed significant enrichment in biological processes, particularly metabolic process and cellular process, which were under positive selection. Notably, seven PSGs (i.e. *ncl*, *srpk1a*, *bach1a*, *rpa1*, *zgpat*, *naf1*, *rad9a*) of *M*. *anguillicaudatus* were involved in “nucleic acid metabolic process” (GO: 0090304), implying that this functional process has played an important role in the adaptation of *M*. *anguillicaudatus*. Previous study^12^ found that the *api5* gene, an anti-apoptotic factor that efficiently suppresses E2F1-induced apoptosis, was under positive selection in diploid *M*. *anguillicaudatus* linage. Likewise, the *api5* was also under positive selection in tetraploid *M*. *anguillicaudatus*, suggesting that anti-apoptosis is critical for the adaptation to various habitat environments of *M*. *anguillicaudatus*. Meanwhile, the endothelial lipase (*lipg*), which is involved in lipid absorption, transport, and metabolism^24^, was positively selected in *M*. *anguillicaudatus* clade. Notably, Yi *et al*.^12^ has reported that the genes (*thrsp* and *srd5a2a*) involved in lipid metabolism closely associated with temperature stress tolerance in fish^25,26^ were under positive selection in diploid *M*. *anguillicaudatus*. It indicates that lipid metabolism also play important roles in adaptation to the complex environments in tetraploid *M*. *anguillicaudatus*. The *garem* and *emp2*, which positively regulate and promote cell proliferation and division^27,28^, were positively selected in the *M*. *anguillicaudatus* clade, and the similar results were also observed in the previous study^12^, suggesting cell proliferation also act as a critical factor in the environmental adaptation in *M*. *anguillicaudatus*.

## Methods

### Ethic Statement

All experimental protocols in this study were approved by the Animal Experimental Ethical Inspection of Laboratory Animal Center, Huazhong Agricultural University, Wuhan, China (HZAUDO-2016-005, 2016-10-26). All efforts were made to minimize the suffering of the animals. All experiments were performed in accordance with relevant guidelines and regulations.

### Sampling and RNA extraction

Healthy adult *M*. *anguillicaudatus* diploid individuals (2n = 50) were collected from the Fisheries Experimental Station of Huazhong Agricultural University and reared in tanks with adequate aeration at 24–26 °C. After anaesthetizing with MS-222 (100 mg/L), 12 tissues (i.e. blood, gill, skin, muscle, liver, spleen, intestine, ovary, testis, kidney, heart and brain) of a male and a female individual were immediately sampled separately. The total RNA of each sample was isolated using TRIzol reagent (TaKaRa, Dalian, China), and then genomic DNA was removed using gDNA eraser (TaKaRa, Dalian, China). RNA quality and concentration were determined using NanoDrop 2000 (Thermo Scientific, DE, USA) and Agilent Bioanalyser 2100 (Agilent Technologies, CA, USA), respectively. Equal amounts of the total RNA from each of the 12 tissues were pooled to generate one sample for library preparation. The first-strand cDNA was synthesized using SMARTer PCR cDNA Synthesis Kit (Clontech, CA, USA). After a round of PCR amplification, the amplified cDNA was size selected into different size fractions to prevent preferential small template sequencing, using the Blue Pippin (Sage Science; MA, USA). Two Iso-Seq libraries (1–5 kb and 4.5–10 kb) were constructed and sequenced on two cells with PacBio Sequel system (PacBio, CA, USA).

### Data processing, isoform clustering

Long reads produced by the PacBio Sequel sequencer were processed with the PacBio IsoSeq pipeline (https://github.com/PacificBiosciences/IsoSeq_SA3nUP) to generate full-length refined consensus transcripts. The reads were filtered using the standard protocols in the SMRT Analysis software suite (https://www.pacb.com/support/software-downloads/), and reads of insert (ROIs) were classified to four categories: full-length non-chimeric, full-length chimeric, non-full-length (non-FL) and short reads. Only reads with two primer and poly-A tail would be classified to full-length in this study. Both full-length non-chimeric reads (FL) and non-full-length reads were used in further analysis. Full-length non-chimeric reads were clustered into consensus. For each cluster, if there were sufficient FL and non-FL coverage, then Quiver was run to polish the consensus. Depending on the Quiver output QV, the script bins the Quiver polished output into either “low QV (LQ)” or “high QV (HQ)”. Only HQ consensus sequences were remained, and the consensus from two libraries were merged and removed the redundancies for the further analysis. Subsequently, we used CD-HIT-EST program^29^ to identify the isoforms based on sequence similarity with parameter: -c 0.98 -T 6 -G 0 -aL 0.90 -AL 100 -aS 0.98 -AS 30.

### Gene annotation and prediction of non-coding RNA identification

Functional annotations were conducted by using BLAST toolkit (E-value ≤ 1 × 10^−5^) against different protein and nucleotide databases of Clusters of Orthologous Groups (COG), Kyoto Encyclopedia of Genes and Genomes (KEGG), a database of conserved Protein families or domains (Pfam), Swiss-prot (a manually annotated, non-redundant protein database), NR (NCBI non-redundant proteins) and nucleotide database (NT). Meanwhile, we used InterProScan5^30^ to obtain the InterPro annotation. Blast2GO^31^ was used to annotate with Gene Ontology (GO) based on the NR annotation. To obtain a high-confidence set of lncRNA genes, we identified lncRNAs from Iso-Seq dataset using a customized pipeline comprised of two steps: (1) removal of annotated protein coding transcripts by BLAST against multiple protein databases together with NCBI Nt database; (2) calculation the coding potential of each transcript using PLEK^32^ (predictor of long non-coding RNAs and messenger RNAs based on an improved k-mer scheme), CNCI^33^ (Coding-Non-Coding Index) and CPAT^34^ (Coding Potential Assessment Tool) to recover the transcripts which can be categorized as noncoding RNAs; (3) removal of potential unannotated protein coding transcripts based on ORF length (>100 aa). The overlapped transcripts identified as non-coding RNAs were remained for the subsequent analysis. The remaining transcripts with known protein domains were excluded by Pfam Scan^35^ according to Hidden Markov Model (HMM). Of these, the transcripts that have more than one exon and which are longer than 200 bases were the potential lncRNAs of *M*. *anguillicaudatus*.

### Assessing transcriptome completeness

A protein set from 248 ultra-conserved Core Eukaryotic Conserved Genes (CEGs) was employed in CEGMA^36^ (Core Eukaryotic Genes Mapping Approach) v2.5 and a set of conserved orthologous proteins was used in BUSCO^37^ (Benchmarking Universal Single-Copy Orthologs), to assess the completeness of the conserved content of these transcripts. The percentages of transcripts that fully aligned and partially aligned to the conserved proteins were calculated.

### Gene family prediction and protein translation

COGENT (coding genome reconstruction tool) v1.3 (https://github.com/Magdoll/Cogent) used *K*-mer similarity profiles to partition full length coding sequences into gene families, after which it reconstructed subreads containing the full coding region. To predict the open reading frames (ORFs) in transcripts, we used the TransDecoder v2.0.1 program (https://transdecoder.github.io/) to define putative coding sequences (CDSs). The predicted CDSs were searched and confirmed by BLASTX (E-value ≤ 1 × 10^−5^) against the protein databases of NR, SWISS-PROT, and KEGG. Those transcripts containing complete ORFs were designated as full-length transcripts.

### Estimation of Ka/Ks in orthologous genes

Coding sequences (CDSs) and proteins of Zebrafish (*Danio rerio*; GRCz10) were downloaded from ENSEMBL database (Release 91). Meanwhile, the transcript data of three *Misgurnus* species, including tetraploid *M*. *anguillicaudatus* (SRR6781483), *M*. *bipartitus* (SRR3744973) and *M*. *mohoity* (SRR3744974), were downloaded from NCBI SRA (Sequence Read Archive) database, and the CDSs and protein sequences of each species were predicted with Transcoder software. The assembly information of these three transcriptomes is listed in Supplementary Table S6. The OrthoFinder algorithm^38^ (version 2.1.2) was utilized to generate orthologous groups of five species for the proteomes datasets (E-value ≤ 1 × 10^−7^). We used PRANK program^39^ to conduct codon alignments for the one to one single copy orthologues with parameter “−iterate = 5”. The regions of ambiguous alignments were excluded using Gblocks^40^ (version 0.91b) with the parameter “−t = c”. Then the alignments with >150 bp (50 codons) were remained for the evolutionary analyses.

The phylogenetic trees were inferred from the concatenated sequences of the orthologous genes (OGs) using the ML method with 1000 bootstraps. We used RAxML^41^ with GTRGAMMA substitution model to perform the ML analysis. The consensus phylogenetic tree was used as the guide tree in the evolutionary analysis. The codeml program implemented in PAML v4.9^42^ with the free-ratio model (model = 1) was selected to calculate the substitution rates of each taxa. When N × *d*_N_ or S × dS < 1 or dS > 1, the orthologues were discarded according to the criterion presented in previous study^43^. The positively selective genes (PSGs) were identified using branch site model following the method described by Yi *et al*.^12^. The significance of LRTs was assessed to discriminate between alternative models. Multiple testing correction was performed with the FDR method. The BEB estimates from each model were used to identify amino acid sites under positive selection when FDR-adjusted *P* value < 0.05. GO enrichment analysis was performed with DAVID Functional Annotation Tool^44^.

### Data availability

The transcript sequences generated in this study were deposited into the figshare website: 10.6084/m9.figshare.6399953.v1.

## Electronic supplementary material

Supplementary Information

## References

[1] Qian X, Ba Y, Zhuang Q, Zhong G (2014). RNA-Seq technology and its application in fish transcriptomics. OMICS.

[2] Schunter C, Vollmer SV, Macpherson E, Pascual M (2014). Transcriptome analyses and differential gene expression in a non-model fish species with alternative mating tactics. BMC genomics.

[3] Gao Z (2012). Transcriptome analysis and SSR/SNP markers information of the blunt snout bream (*Megalobrama amblycephala*). Plos one.

[4] Chen Z (2008). Transcriptomic and genomic evolution under constant cold in Antarctic notothenioid fish. Proc. Natl. Acad. Sci..

[5] Grabherr MG (2011). Full-length transcriptome assembly from RNA-Seq data without a reference genome. Nat. Biotechnol..

[6] Rhoads A, Au KF (2015). PacBio sequencing and its applications. GPB.

[7] Weirather JL (2017). Comprehensive comparison of Pacific Biosciences and Oxford Nanopore Technologies and their applications to transcriptome analysis. F1000Research.

[8] Chen J, Zhu S (1984). Phylogenetic relationships of the subfamily in the loach family Cobitidae (Pisces). Acta Zootax. Sin..

[9] Feng B, Soojin VY, Li R, Zhou X (2017). Comparison of age and growth performance of diploid and tetraploid loach *Misgurnus anguillicaudatus* in the Yangtze River basin, China. Environ. Biol. Fish..

[10] Zhou X (2014). Comparative analysis of mitochondrial genomes in distinct nuclear ploidy loach *Misgurnus anguillicaudatus* and its implications for polyploidy evolution. Plos one.

[11] Yi S, Li Y, Wang W (2017). Selection shapes the patterns of codon usage in three closely related species of genus *Misgurnus*. Genomics.

[12] Yi S, Wang S, Zhong J, Wang W (2016). Comprehensive Transcriptome Analysis Provides Evidence of Local Thermal Adaptation in Three Loaches (Genus: Misgurnus). Int. J. Mol. Sci..

[13] Yi S, Zhong J, Huang S, Wang S, Wang W (2017). Morphological comparison and DNA barcoding of four closely related species in the genera *Misgurnus* and *Paramisgurnus* (Cypriniformes: Cobitidae). Biochem. Syst. Ecol..

[14] Nawrocki EP (2014). Rfam 12.0: updates to the RNA families database. Nucleic Acids Res..

[15] Li J (2017). Long read reference genome-free reconstruction of a full-length transcriptome from Astragalus membranaceus reveals transcript variants involved in bioactive compound biosynthesis. Cell discovery.

[16] Xie Y (2014). SOAPdenovo-Trans: de novo transcriptome assembly with short RNA-Seq reads. Bioinformatics.

[17] Zerbino DR, Birney E (2008). Velvet: algorithms for de novo short read assembly using de Bruijn graphs. Genome Res..

[18] Luo W (2016). Developmental transcriptome analysis and identification of genes involved in formation of intestinal air-breathing function of Dojo loach, *Misgurnus anguillicaudatus*. Sci. Rep..

[19] Huang S, Cao X, Tian X (2016). Transcriptomic analysis of compromise between air-breathing and nutrient uptake of posterior intestine in loach (*Misgurnus anguillicaudatus*), an air-breathing fish. Mar. Biotechnol..

[20] Long Y (2013). De novo assembly of mud loach (*Misgurnus anguillicaudatus*) skin transcriptome to identify putative genes involved in immunity and epidermal mucus secretion. Plos one.

[21] Li, Y. *et al*. A study on the distribution of polyploid loaches in China. Nippon Suisan Gakkaishi (Japan) (2008).

[22] Cui L (2013). First record of the natural occurrence of pentaploid loach, Misgurnus anguillicaudatus in Hubei Province, China. Folia Zool..

[23] Abbas K, Li MY, Wang WM, Zhou XY (2009). First record of the natural occurrence of hexaploid loach *Misgurnus anguillicaudatus* in Hubei Province, China. J. Fish Biol..

[24] Mank-Seymour AR, Durham KL, Thompson JF, Seymour AB, Milos PM (2004). Association between single-nucleotide polymorphisms in the endothelial lipase (LIPG) gene and high-density lipoprotein cholesterol levels. BBA-Mol.Cell Biol. L..

[25] Gracey AY (2004). Coping with cold: An integrative, multitissue analysis of the transcriptome of a poikilothermic vertebrate. Proc. Natl. Acad. Sci. USA.

[26] Hu JW (2014). Transcriptional responses of olive flounder (*Paralichthys olivaceus*) to low temperature. Plos One.

[27] Taniguchi T (2013). A brain-specific Grb2-associated regulator of extracellular signal-regulated kinase (Erk)/mitogen-activated protein kinase (MAPK)(GAREM) subtype, GAREM2, contributes to neurite outgrowth of neuroblastoma cells by regulating Erk signaling. J. Biol. Chem..

[28] Chung LK (2017). Epithelial membrane protein 2: Molecular interactions and clinical implications. J. Clin. Neurosci..

[29] Fu L, Niu B, Zhu Z, Wu S, Li W (2012). CD-HIT: accelerated for clustering the next-generation sequencing data. Bioinformatics.

[30] Jones P (2014). InterProScan 5: genome-scale protein function classification. Bioinformatics.

[31] Conesa, A. & Götz, S. Blast2GO: A comprehensive suite for functional analysis in plant genomics. Int. J. Plant Genomics 2008 (2008). 10.1155/2008/619832 PMC2375974 18483572

[32] Li A, Zhang J, Zhou Z (2014). PLEK: a tool for predicting long non-coding RNAs and messenger RNAs based on an improved k-mer scheme. BMC bioinformatics.

[33] Sun L (2013). Utilizing sequence intrinsic composition to classify protein-coding and long non-coding transcripts. Nucleic Acids Res..

[34] Wang L (2013). CPAT: Coding-Potential Assessment Tool using an alignment-free logistic regression model. Nucleic Acids Res..

[35] Finn RD (2015). The Pfam protein families database: towards a more sustainable future. Nucleic Acids Res..

[36] Parra G, Bradnam K, Korf I (2007). CEGMA: a pipeline to accurately annotate core genes in eukaryotic genomes. Bioinformatics.

[37] Simão FA, Waterhouse RM, Ioannidis P, Kriventseva EV, Zdobnov EM (2015). BUSCO: assessing genome assembly and annotation completeness with single-copy orthologs. Bioinformatics.

[38] Emms DM, Kelly S (2015). OrthoFinder: solving fundamental biases in whole genome comparisons dramatically improves orthogroup inference accuracy. Genome Biol..

[39] Löytynoja A, Goldman N (2008). Phylogeny-aware gap placement prevents errors in sequence alignment and evolutionary analysis. Science.

[40] Castresana J (2000). Selection of conserved blocks from multiple alignments for their use in phylogenetic analysis. Mol. Biol. Evol..

[41] Stamatakis A (2014). RAxML version 8: a tool for phylogenetic analysis and post-analysis of large phylogenies. Bioinformatics.

[42] Yang Z (2007). PAML 4: phylogenetic analysis by maximum likelihood. Mol. Biol. Evol..

[43] Goodman M (2009). Phylogenomic analyses reveal convergent patterns of adaptive evolution in elephant and human ancestries. Proc. Natl. Acad. Sci. USA.

[44] Huang DW, Sherman BT, Lempicki RA (2008). Systematic and integrative analysis of large gene lists using DAVID bioinformatics resources. Nat. Protoc..

