# Supplementary material for: Full-length transcriptome of Misgurnus anguillicaudatus provides insights into evolution of genus Misgurnus
Source: Sci Rep. 2018 Aug 3;8:11699. doi: 10.1038/s41598-018-29991-6 (PMC6076316; doi:10.1038/s41598-018-29991-6)
Supplement: Supplementary file 1 — Supplementary Information [file 41598_2018_29991_MOESM1_ESM.zip › Supplementary Table S3.docx]

**Full-length transcriptome of *Misgurnus anguillicaudatus* provides insights into evolution of genus *Misgurnus***

Shaokui Yi^1, 2^, Xiaoyun Zhou^1*^, Jie Li^1^, Manman Zhang^1^ & Shuangshuang Luo^1^

^1^ College of Fisheries, Key Lab of Freshwater Animal Breeding, Ministry of Agriculture, Huazhong Agricultural University, Wuhan, 430070, P.R. China

^2^ Fish Genetics and Breeding Laboratory, the Ohio State University South Centers, Piketon 45661, USA

**Table S3 The Rfam annotations of lncRNAs identified in *M. anguillicaudatus***

| Transcript ID | Length | Rank | E-value | score | Model name | Start | End | strand | GC content |
| --- | --- | --- | --- | --- | --- | --- | --- | --- | --- |
| [2n]_72537 | 1355 | 1 | 1.10E-07 | 40.3 | MIR167_1 | 528 | 853 | + | 0.42 |
| [2n]_72537 | 1355 | 2 | 1.20E-07 | 40.2 | MIR167_1 | 854 | 529 | - | 0.42 |
| [2n]_39834 | 2980 | 1 | 9.10E-03 | 28.6 | UnaL2 | 2802 | 2749 | - | 0.24 |
| [2n]_51872 | 2361 | 1 | 3.00E-04 | 32.3 | WsnRNA46 | 271 | 422 | + | 0.35 |
| [2n]_71213 | 1455 | 1 | 8.90E-03 | 26.7 | EF0605_EF0606 | 844 | 710 | - | 0.3 |
| [2n]_67638 | 1672 | 1 | 5.10E-03 | 25.6 | mir-996 | 581 | 491 | - | 0.44 |
| [2n]_72615 | 1349 | 1 | 1.90E-04 | 30.9 | MIR821 | 32 | 168 | + | 0.35 |
| [2n]_43840 | 2745 | 1 | 8.10E-03 | 27.1 | MIR403 | 910 | 1022 | + | 0.55 |
| [2n]_49899 | 2446 | 1 | 2.10E-06 | 43.1 | MIR530 | 1132 | 1344 | + | 0.31 |
| [2n]_49899 | 2446 | 2 | 8.30E-06 | 40.6 | MIR530 | 1344 | 1132 | - | 0.31 |
| [2n]_74911 | 1085 | 1 | 5.10E-03 | 21.4 | SCARNA7 | 1 | 106 | + | 0.4 |
| [2n]_73836 | 1232 | 1 | 7.30E-04 | 31.3 | UnaL2 | 890 | 942 | + | 0.34 |
| [2n]_67982 | 1654 | 1 | 4.10E-03 | 32.1 | MIR820 | 530 | 657 | + | 0.55 |
| [2n]_47194 | 2569 | 1 | 2.30E-06 | 48.1 | SNORD42 | 22 | 87 | + | 0.36 |
| [2n]_64231 | 1840 | 1 | 6.90E-04 | 28.5 | mir-42 | 976 | 1069 | + | 0.53 |
| [2n]_37311 | 3159 | 1 | 2.80E-03 | 28.3 | mir-395 | 1334 | 1446 | + | 0.35 |
| [2n]_76462 | 585 | 1 | 7.10E-08 | 42 | mir-87 | 251 | 370 | + | 0.43 |
| [2n]_76462 | 585 | 2 | 7.10E-08 | 42 | mir-87 | 364 | 245 | - | 0.43 |
| [2n]_71695 | 1421 | 1 | 8.60E-03 | 24.8 | mir-160 | 454 | 595 | + | 0.55 |
| [2n]_76710 | 391 | 1 | 1.40E-14 | 66.6 | WsnRNA46 | 136 | 282 | + | 0.3 |
| [2n]_76710 | 391 | 2 | 9.40E-05 | 30.3 | mir-34 | 265 | 156 | - | 0.31 |
| [2n]_69107 | 1590 | 1 | 3.90E-07 | 42 | WsnRNA46 | 632 | 756 | + | 0.37 |
| [2n]_69107 | 1590 | 2 | 1.80E-05 | 37.5 | MIR2118 | 640 | 749 | + | 0.34 |

**Continue**

| [2n]_65359 | 1787 | 1 | 3.40E-06 | 39.6 | mir-616 | 771 | 877 | + | 0.37 |
| --- | --- | --- | --- | --- | --- | --- | --- | --- | --- |
| [2n]_65359 | 1787 | 2 | 1.90E-03 | 29.1 | mir-63 | 867 | 774 | - | 0.38 |
| [2n]_61959 | 1933 | 1 | 4.70E-04 | 30.9 | mir-9 | 973 | 1026 | + | 0.39 |
| [2n]_61959 | 1933 | 2 | 2.00E-03 | 28.6 | mir-9 | 1026 | 973 | - | 0.39 |
| [2n]_58363 | 2079 | 1 | 1.70E-03 | 24.6 | rli43 | 734 | 801 | + | 0.26 |
| [2n]_75198 | 1032 | 1 | 2.30E-04 | 30.1 | MIR821 | 141 | 3 | - | 0.35 |
| [2n]_57521 | 2115 | 1 | 1.10E-03 | 29.4 | MIR474 | 1196 | 1279 | + | 0.45 |
| [2n]_68992 | 1598 | 1 | 3.50E-03 | 26.9 | mir-395 | 835 | 943 | + | 0.27 |
| [2n]_62109 | 1928 | 1 | 6.80E-05 | 30.7 | mir-203 | 736 | 819 | + | 0.43 |
| [2n]_62109 | 1928 | 2 | 9.40E-05 | 34.3 | mir-306 | 804 | 890 | + | 0.51 |
| [2n]_70926 | 1476 | 1 | 2.70E-07 | 40.7 | mir-393 | 619 | 487 | - | 0.45 |
| [2n]_21585 | 5340 | 1 | 6.80E-06 | 41.6 | UnaL2 | 2068 | 2015 | - | 0.3 |
| [2n]_57563 | 2113 | 1 | 6.30E-07 | 44.1 | UnaL2 | 1873 | 1926 | + | 0.33 |
| [2n]_53350 | 2297 | 1 | 6.10E-03 | 27.5 | mir-1 | 597 | 661 | + | 0.46 |
| [2n]_67063 | 1703 | 1 | 1.20E-06 | 38.5 | MIR477 | 116 | 14 | - | 0.64 |
| [2n]_73023 | 1314 | 1 | 9.40E-03 | 31.6 | mir-149 | 650 | 722 | + | 0.7 |
| [2n]_57218 | 2129 | 1 | 3.40E-03 | 28.4 | WsnRNA46 | 1109 | 1006 | - | 0.25 |
| [2n]_66616 | 1725 | 1 | 4.40E-03 | 18.7 | DLX6-AS1_2 | 797 | 693 | - | 0.32 |
| [2n]_73373 | 1280 | 1 | 4.20E-03 | 19.2 | NRON | 242 | 417 | + | 0.34 |
| [2n]_52394 | 2337 | 1 | 1.90E-03 | 21 | snoR122 | 323 | 286 | - | 0.24 |
| [2n]_49783 | 2451 | 1 | 3.50E-03 | 28.5 | SCARNA11 | 1494 | 1399 | - | 0.35 |
| [2n]_63308 | 1879 | 1 | 4.00E-04 | 33.1 | UnaL2 | 1680 | 1627 | - | 0.35 |
| [2n]_67885 | 1659 | 1 | 2.90E-04 | 27.1 | SCARNA7 | 628 | 479 | - | 0.41 |
| [2n]_60253 | 2002 | 1 | 2.90E-03 | 26.7 | mir-996 | 391 | 527 | + | 0.46 |
| [2n]_76446 | 591 | 1 | 5.20E-03 | 22.1 | tsr24 | 543 | 418 | - | 0.29 |
| [2n]_70149 | 1527 | 1 | 1.60E-04 | 32.1 | mir-558 | 923 | 859 | - | 0.4 |
| [2n]_70149 | 1527 | 2 | 9.90E-03 | 20.8 | SCARNA7 | 1031 | 887 | - | 0.39 |
| [2n]_42670 | 2813 | 1 | 8.80E-06 | 37.5 | mir-576 | 1860 | 1952 | + | 0.43 |
| [2n]_44580 | 2707 | 1 | 3.80E-05 | 37.6 | UnaL2 | 906 | 853 | - | 0.39 |
| [2n]_74253 | 1181 | 1 | 7.20E-10 | 52.7 | MIR821 | 722 | 989 | + | 0.26 |
| [2n]_63320 | 1878 | 1 | 3.10E-03 | 28.7 | mir-616 | 313 | 399 | + | 0.41 |
| [2n]_76708 | 393 | 1 | 3.20E-36 | 158 | MIR821 | 92 | 333 | + | 0.33 |
| [2n]_76708 | 393 | 2 | 4.70E-08 | 44.2 | mir-355 | 268 | 160 | - | 0.32 |
| [2n]_37471 | 3146 | 1 | 3.00E-03 | 30.5 | UnaL2 | 851 | 799 | - | 0.38 |
| [2n]_75185 | 1035 | 1 | 2.60E-09 | 48.3 | MIR1222 | 621 | 439 | - | 0.49 |
| [2n]_75185 | 1035 | 2 | 2.60E-09 | 48.3 | MIR1222 | 441 | 623 | + | 0.49 |
| [2n]_63882 | 1855 | 1 | 3.40E-05 | 37.8 | mir-653 | 60 | 126 | + | 0.37 |
| [2n]_63882 | 1855 | 2 | 3.40E-05 | 37.8 | mir-653 | 463 | 529 | + | 0.37 |
| [2n]_69616 | 1561 | 1 | 1.90E-04 | 34 | UnaL2 | 565 | 512 | - | 0.37 |
| [2n]_62546 | 1909 | 1 | 2.10E-08 | 49.6 | UnaL2 | 999 | 946 | - | 0.33 |
| [2n]_63138 | 1885 | 1 | 2.80E-04 | 30 | mir-280 | 1717 | 1813 | + | 0.27 |

**Continue**

| [2n]_53766 | 2278 | 1 | 8.80E-03 | 26.7 | 5S_rRNA | 373 | 466 | + | 0.59 |
| --- | --- | --- | --- | --- | --- | --- | --- | --- | --- |
| [2n]_66735 | 1718 | 1 | 2.50E-03 | 24.8 | EBv-sisRNA-1 | 1401 | 1481 | + | 0.44 |
| [2n]_66708 | 1720 | 1 | 6.10E-03 | 26.1 | mir-395 | 430 | 315 | - | 0.28 |
| [2n]_64340 | 1836 | 1 | 1.20E-03 | 29.5 | MIR403 | 167 | 357 | + | 0.31 |
| [2n]_76517 | 551 | 1 | 2.70E-06 | 35.9 | mir-1253 | 234 | 352 | + | 0.55 |
| [2n]_76517 | 551 | 2 | 7.10E-05 | 30 | mir-767 | 336 | 245 | - | 0.57 |
| [2n]_73558 | 1262 | 1 | 7.20E-03 | 30.8 | RsaH | 938 | 812 | - | 0.31 |
| [2n]_70417 | 1511 | 1 | 8.50E-03 | 21.1 | SCARNA7 | 292 | 433 | + | 0.32 |
| [2n]_60370 | 1997 | 1 | 5.70E-03 | 25.9 | mir-652 | 773 | 867 | + | 0.46 |
| [2n]_71728 | 1419 | 1 | 4.30E-03 | 25.2 | mir-85 | 358 | 454 | + | 0.38 |
| [2n]_65823 | 1765 | 1 | 8.50E-07 | 41.9 | IRE_II | 941 | 971 | + | 0.35 |
| [2n]_65823 | 1765 | 2 | 6.50E-06 | 38.8 | IRE_II | 829 | 859 | + | 0.45 |
| [2n]_69240 | 1582 | 1 | 9.10E-05 | 33.4 | MIR403 | 209 | 316 | + | 0.53 |
| [2n]_69240 | 1582 | 2 | 3.60E-03 | 26.9 | MIR1428 | 130 | 244 | + | 0.49 |
| [2n]_49524 | 2463 | 1 | 5.40E-09 | 42.8 | EF0820_EF0821 | 1149 | 1360 | + | 0.36 |
| [2n]_49524 | 2463 | 2 | 5.20E-08 | 39.4 | EF0820_EF0821 | 1346 | 1135 | - | 0.36 |
| [2n]_62181 | 1925 | 1 | 6.20E-05 | 36.2 | UnaL2 | 40 | 1 | - | 0.43 |
| [2n]_56968 | 2140 | 1 | 7.20E-06 | 37 | MIR811 | 1891 | 1670 | - | 0.45 |
| [2n]_57470 | 2117 | 1 | 3.60E-03 | 29.6 | UnaL2 | 509 | 560 | + | 0.29 |
| [2n]_63896 | 1854 | 1 | 3.80E-03 | 28 | WsnRNA46 | 1004 | 891 | - | 0.28 |
| [2n]_75385 | 991 | 1 | 6.50E-03 | 24.4 | mir-14 | 426 | 392 | - | 0.43 |
| [2n]_76093 | 784 | 1 | 4.70E-06 | 39 | UnaL2 | 135 | 188 | + | 0.35 |
| [2n]_56117 | 2175 | 1 | 6.10E-03 | 20 | CrcZ | 2010 | 1957 | - | 0.28 |
| [2n]_72682 | 1343 | 1 | 3.50E-10 | 51.8 | MIR390 | 638 | 745 | + | 0.36 |
| [2n]_72682 | 1343 | 2 | 1.10E-08 | 46.5 | MIR390 | 745 | 638 | - | 0.36 |
| [2n]_69333 | 1576 | 1 | 6.80E-03 | 25.8 | mir-395 | 262 | 146 | - | 0.29 |
| [2n]_59540 | 2029 | 1 | 5.40E-03 | 22.4 | SCARNA7 | 1 | 92 | + | 0.43 |
| [2n]_70566 | 1501 | 1 | 3.30E-03 | 25.9 | SNORD46 | 1410 | 1339 | - | 0.49 |
| [2n]_68616 | 1619 | 1 | 6.40E-03 | 22.2 | TeloSII_ncR43 | 211 | 386 | + | 0.32 |
| [2n]_69753 | 1552 | 1 | 1.10E-03 | 28.1 | MIR821 | 1019 | 1142 | + | 0.38 |
| [2n]_75541 | 956 | 1 | 7.80E-11 | 58.5 | mir-578 | 443 | 538 | + | 0.38 |
| [2n]_75541 | 956 | 2 | 7.80E-11 | 58.5 | mir-578 | 544 | 449 | - | 0.38 |
| [2n]_55852 | 2185 | 1 | 7.10E-03 | 18.9 | snoR118 | 1101 | 1040 | - | 0.32 |
| [2n]_66512 | 1729 | 1 | 4.60E-07 | 44.3 | UnaL2 | 320 | 372 | + | 0.3 |
| [2n]_53393 | 2294 | 1 | 4.90E-04 | 33.7 | mir-653 | 5 | 68 | + | 0.33 |
| [2n]_43755 | 2751 | 1 | 8.70E-08 | 44.4 | MIR811 | 334 | 500 | + | 0.44 |
| [2n]_43755 | 2751 | 2 | 6.40E-06 | 36.1 | mir-996 | 1030 | 1131 | + | 0.4 |
| [2n]_37787 | 3121 | 1 | 2.10E-03 | 22.1 | CrcZ | 1316 | 1397 | + | 0.29 |
| [2n]_61794 | 1940 | 1 | 4.50E-07 | 41.3 | MIR811 | 1052 | 871 | - | 0.41 |
| [2n]_61794 | 1940 | 2 | 5.10E-06 | 37.4 | MIR811 | 871 | 1052 | + | 0.41 |
| [2n]_33572 | 3504 | 1 | 8.90E-03 | 25 | MIR167_1 | 1285 | 1195 | - | 0.27 |
