# Supplementary material for: Full-length transcriptome of Misgurnus anguillicaudatus provides insights into evolution of genus Misgurnus
Source: Sci Rep. 2018 Aug 3;8:11699. doi: 10.1038/s41598-018-29991-6 (PMC6076316; doi:10.1038/s41598-018-29991-6)
Supplement: Supplementary file 1 — Supplementary Information [file 41598_2018_29991_MOESM1_ESM.zip › Supplementary Table S5.docx]

**Full-length transcriptome of *Misgurnus anguillicaudatus* provides insights into evolution of genus *Misgurnus***

Shaokui Yi^1, 2^, Xiaoyun Zhou^1*^, Jie Li^1^, Manman Zhang^1^ & Shuangshuang Luo^1^

^1^ College of Fisheries, Key Lab of Freshwater Animal Breeding, Ministry of Agriculture, Huazhong Agricultural University, Wuhan, 430070, P.R. China

^2^ Fish Genetics and Breeding Laboratory, the Ohio State University South Centers, Piketon 45661, USA

**Table S5** The positively selected genes identified in *M. anguillicaudatus* clade using branch model of codeml

| Orthologues | *df* | lnL A null | lnL model A | FDR | Gene ID (Zebrafish) | Gene name | Gene description |
| --- | --- | --- | --- | --- | --- | --- | --- |
| OG0022 | 1 | -790.4406 | -787.8886 | 0.0336 | ENSDARP00000111133 | *si:dkey-54n8.2* | si:dkey-54n8.2 |
| OG0102 | 1 | -4800.2492 | -4796.3651 | 0.0206 | ENSDARP00000082748 | *WASHC4* | WASH complex subunit 4 |
| OG0154 | 1 | -2845.1614 | -2840.6578 | 0.0119 | ENSDARP00000082031 | *col6a2* | collagen, type VI, alpha 2 |
| OG0223 | 1 | -2670.8246 | -2677.5536 | 0.0025 | ENSDARP00000119939 | *si:ch211-284e13.5* | si:ch211-284e13.5 |
| OG0306 | 1 | -4445.6432 | -4442.7741 | 0.0286 | ENSDARP00000024393 | *garem* | GRB2 associated, regulator of MAPK1 |
| OG0395 | 1 | -4366.8728 | -4363.4411 | 0.0237 | ENSDARP00000141227 | *si:ch211-71n6.4* | si:ch211-71n6.4 |
| OG0501 | 1 | -1819.3677 | -1816.2824 | 0.0237 | ENSDARP00000111815 | *ncl* | nucleolin |
| OG0507 | 1 | -2475.0576 | -2472.8419 | 0.0365 | ENSDARP00000060033 | *zgc:113149* | zgc:113149 |

**Continue**

| OG0547 | 1 | -3423.0609 | -3420.4611 | 0.0336 | ENSDARP00000032398 | *srpk1a* | SRSF protein kinase 1a |
| --- | --- | --- | --- | --- | --- | --- | --- |
| OG0583 | 1 | -2943.1965 | -2940.7808 | 0.0343 | ENSDARP00000126600 | *api5* | apoptosis inhibitor 5 |
| OG0614 | 1 | -4152.1066 | -4149.5132 | 0.0336 | ENSDARP00000127728 | *bach1a* | BTB and CNC homology 1, basic leucine zipper transcription factor 1 a |
| OG0692 | 1 | -3899.0677 | -3896.6763 | 0.0343 | ENSDARP00000020784 | *tbc1d17* | TBC1 domain family, member 17 |
| OG0759 | 1 | -2704.5688 | -2697.5599 | 0.0025 | ENSDARP00000029067 | *rpa1* | replication protein A1 |
| OG0884 | 1 | -3311.5977 | -3308.9414 | 0.0336 | ENSDARP00000089620 | *lipg* | lipase, endothelial |
| OG1006 | 1 | -1606.2151 | -1601.4630 | 0.0115 | ENSDARP00000114381 | *nfu1* | NFU1 iron-sulfur cluster scaffold homolog (S. cerevisiae) |
| OG1009 | 1 | -4523.1269 | -4520.8970 | 0.0365 | ENSDARP00000119903 | *serping1* | serpin peptidase inhibitor, clade G (C1 inhibitor), member 1 |
| OG1052 | 1 | -1471.8439 | -1461.6056 | 0.0002 | ENSDARP00000055236 | *ramp2* | receptor (G protein-coupled) activity modifying protein 2 |
| OG1164 | 1 | -1076.7865 | -1074.3671 | 0.0343 | ENSDARP00000065494 | *emp2* | epithelial membrane protein 2 |
| OG1167 | 1 | -3332.0298 | -3329.7640 | 0.0365 | ENSDARP00000010750 | *cwf19l1* | CWF19-like 1, cell cycle control |
| OG1175 | 1 | -1745.7470 | -1742.5547 | 0.0237 | ENSDARP00000088818 | *gas1b* | growth arrest-specific 1b |
| OG1178 | 1 | -2436.9142 | -2433.7157 | 0.0237 | ENSDARP00000030077 | *zgpat* | zinc finger, CCCH-type with G patch domain |
| OG1413 | 1 | -2847.4668 | -2845.2146 | 0.0365 | ENSDARP00000114221 | *naf1* | nuclear assembly factor 1 homolog (S. cerevisiae) |
| OG1508 | 1 | -2803.6829 | -2800.3995 | 0.0237 | ENSDARP00000103358 | *serpina1* | serpin peptidase inhibitor, clade A (alpha-1 antiproteinase, antitrypsin), member 1 |

**Continue**

| OG1578 | 1 | -2065.5302 | -2063.5090 | 0.0444 | ENSDARP00000109435 | *pomk* | protein-O-mannose kinase |
| --- | --- | --- | --- | --- | --- | --- | --- |
| OG1612 | 1 | -1664.5054 | -1660.9996 | 0.0237 | ENSDARP00000054419 | *rad9a* | RAD9 checkpoint clamp component A |
| OG1652 | 1 | -745.6482 | -743.2575 | 0.0343 | ENSDARP00000022759 | *sf3b4* | splicing factor 3b, subunit 4 |
| OG1728 | 1 | -1124.2196 | -1121.0987 | 0.0237 | ENSDARP00000135138 | *ier2a* | immediate early response 2a |
| OG1810 | 1 | -1155.6478 | -1152.3680 | 0.0237 | ENSDARP00000086688 | *mrpl9* | mitochondrial ribosomal protein L9 |
