# Supplementary material for: Full-length transcriptome of Misgurnus anguillicaudatus provides insights into evolution of genus Misgurnus
Source: Sci Rep. 2018 Aug 3;8:11699. doi: 10.1038/s41598-018-29991-6 (PMC6076316; doi:10.1038/s41598-018-29991-6)
Supplement: Supplementary file 1 — Supplementary Information [file 41598_2018_29991_MOESM1_ESM.zip › Supplementary Table S6.docx]

**Full-length transcriptome of *Misgurnus anguillicaudatus* provides insights into evolution of genus *Misgurnus***

Shaokui Yi^1, 2^, Xiaoyun Zhou^1*^, Jie Li^1^, Manman Zhang^1^ & Shuangshuang Luo^1^

^1^ College of Fisheries, Key Lab of Freshwater Animal Breeding, Ministry of Agriculture, Huazhong Agricultural University, Wuhan, 430070, P.R. China

^2^ Fish Genetics and Breeding Laboratory, the Ohio State University South Centers, Piketon 45661, USA

**Table S6 The assembly information of three *Misgurnus* species**

| Species name | SRA accession number | Unigene number | Mean Length (bp) | Unigene N50 | Reference |
| --- | --- | --- | --- | --- | --- |
| *M. anguillicaudatus* (tetraploid) | SRR6781483 | 52,576 | 907 | 1623 |  |
| *M. bipartitus* | SRR3744973 | 84,887 | 864 | 1831 | Yi et al^1^ |
| *M. mohoity* | SRR3744974 | 78,707 | 842 | 1677 | Yi et al^1^ |

**Reference**

1. Yi, S., Wang, S., Zhong, J. & Wang, W. Comprehensive Transcriptome Analysis Provides Evidence of Local Thermal Adaptation in Three Loaches (Genus: *Misgurnus*). *Int. J. Mol. Sci.* **17**, 1943 (2016)
